# Associations of postpartum mother-infant bonding with maternal childhood maltreatment and postpartum mental health: a cross-sectional study

**DOI:** 10.1186/s12884-019-2426-0

**Published:** 2019-08-05

**Authors:** Franziska Lehnig, Michaela Nagl, Holger Stepan, Birgit Wagner, Anette Kersting

**Affiliations:** 1 IFB AdiposityDiseases, Leipzig University Medical Center, Philipp-Rosenthal-Straße 27, 04103 Leipzig, Germany; 2 Department of Psychosomatic Medicine and Psychotherapy, University of Leipzig, Semmelweisstraße 10, 04103 Leipzig, Germany; 3 Department of Obstetrics, University of Leipzig, Liebigstraße 20a, 04103 Leipzig, Germany; 4 Department of Clinical Psychology and Psychotherapy, MSB Medical School Berlin, Calandrellistraße 1-9, 12247 Berlin, Germany

**Keywords:** Mother-infant bonding, Childhood maltreatment, Depression, Anxiety, Postpartum

## Abstract

**Background:**

After delivery, some women experience impairment of their mother-infant bonding (MIB), which can lead to long-term disturbances of the mother-child relationship and the child’s social-emotional development. Little is known about the association between early maternal bonding problems and mothers’ own adverse childhood experiences, even though the hypothesis of the intergenerational transmission of caregiving indicates continuity in parenting quality across generations. Therefore, the current study aimed at examining the relationship between maternal childhood maltreatment and postpartum MIB, controlling for the role of postpartum mental health.

**Methods:**

From February 2014 to March 2015, 725 women completed self-report measures 2 months postpartum. Maternal childhood maltreatment was assessed with the Childhood Trauma Questionnaire, postpartum depression with the Revised Beck Depression Inventory, postpartum anxiety with the Symptom Checklist-90-Revised, and postpartum MIB with the abridged version of the Postpartum Bonding Questionnaire. Data were analysed using a hierarchical regression analysis.

**Results:**

Almost 46% of the included women reported at least one type of childhood maltreatment with emotional neglect being most prevalent. 13% displayed at least mild postpartum depressive symptomatology and 20% scored above the 75^th^ percentile for postpartum anxiety. In the final regression model, which explained 29% of variance, higher severity of maternal emotional neglect in childhood, higher levels of postpartum depression and higher education were significantly related to more postpartum MIB impairment. In contrast, higher severity of maternal physical neglect was significantly associated with less postpartum MIB impairment.

**Conclusions:**

This study is the first to explore the relationship between diverse types of maternal childhood maltreatment and postpartum MIB, adjusting for postpartum mental health. Maternal experiences of emotional neglect and postpartum depressive symptoms could serve as indicators to identify and support mothers with heightened risk for bonding problems, but results need to be validated in longitudinal studies.

## Background

The development of a healthy bond with the newborn represents a central psychological process for the mother in the postpartum period [1]. Mother-infant bonding (MIB) is defined as an affective state of the mother including maternal emotions towards the infant, and considered to be the basis for the child’s later attachment and sense of self [2]. Although the majority of women have no difficulties developing an affectionate relationship with their newborn, disturbances of the postpartum MIB are present in 7% [3] to 11.3% [4] of women in the general population. The prevalence of disorders of the mother-infant relationship in clinical samples is even higher, with up to 25% of women [1] reporting a lack of or delay in emotional response to the infant, feelings of irritability, hostility, aggressive impulses or rejection of the child [5–7]. Disturbances of the MIB can lead to long-term impairment of the mother-child relationship including child maltreatment [8], and increase the child’s risk of developing psychopathology in adulthood [9]. Given the important effects of postpartum MIB on the future health and development of the infant and mother, it is important to investigate factors interfering with the quality of MIB in order to early identify and support mothers at heightened risk for bonding problems.

### Maternal childhood maltreatment

In the past, research on mother-infant relationship has focused on maternal factors like a mother’s own upbringing experiences. In this context, the hypothesis of the intergenerational transmission of caregiving has emerged, indicating continuity in parenting quality across generations [10, 11]. In particular, women with a history of childhood maltreatment show impairment in their adaption to parenthood and family life [12, 13]. Childhood maltreatment is defined as “the abuse and neglect that occurs to children under 18 years of age.” [14]. Five types of maltreatment are widely recognised, namely sexual, physical, and emotional abuse as well as physical and emotional neglect. Sexual abuse is defined as “any completed or attempted sexual act, sexual contact, or non-contact sexual interaction with a child by a caregiver” ([15], p. 69). Physical abuse is defined as “intentional use of physical force or implements against a child that results in, or has the potential to result in, physical injury” ([15], p. 69). Emotional abuse is defined as “intentional behaviour that conveys to a child that he/she is worthless, flawed, unloved, unwanted, endangered, or valued only in meeting another’s needs” ([15], p. 69). Physical neglect refers to the failure to meet a child’s physical needs (e.g., failure to provide adequate nutrition and clothing), whereas emotional neglect refers to the failure to meet a child’s emotional needs (e.g., failure to provide adequate nurturance and affection) [16]. Women who have experienced childhood maltreatment are more likely to exhibit an intrusive parenting style [17], anxiety about intimate parenting [18] and punitive behaviour [19, 20], and to abuse their own offspring [21] (see [22] for review). Furthermore, a recent meta-analysis showed a tendency that parenting differs depending on the type of maltreatment mothers experienced as a child [13]. In detail, the negative effect of emotional and/or physical abuse on parenting was greater than that of sexual abuse. Experiences of childhood neglect were not included in this analysis due to a lack of adequate studies.

While the adverse effects of childhood maltreatment on general parenting outcomes are well established, only a few studies have addressed its influence on early postpartum MIB. Available evidence suggests that a maternal history of childhood maltreatment is not per se related to MIB disturbances across the first year postpartum, but mediated by postpartum psychopathology of the mother, e.g., postpartum depression and posttraumatic stress disorder (PTSD) [23–26]. The existing studies used composite measures of childhood maltreatment, which do not differentiate between diverse types of maltreatment. Therefore, little is known about specific effects of different types of childhood maltreatment on postpartum MIB. To date, only one study focused separately on maternal emotional abuse in childhood as an important predictor of postpartum MIB disturbances in a clinical sample of mothers [27]. However, as traumatic childhood experiences are interrelated and tend to co-occur [15, 28, 29], it is necessary to include different types of maltreatment in order to assess to what extent other maltreatment forms may account for this association. In particular, childhood neglect has been understudied despite its high prevalence and negative consequences [16]. The current study addresses some research gaps by examining the differential effects of different types of maternal childhood maltreatment on postpartum MIB taking into account their frequent co-occurrence.

### Maternal mental health

The risk for mental disorders in women’s lives increases during the challenging period of pregnancy and puerperium [30]. Referring to a systematic review, up to 19.2% of women suffer from a major or minor depression during the first 3 months after delivery [31]. The prevalence of anxiety disorders among postpartum women is comparably high with estimates between 8% and 13% [32, 33]. The mental health conditions do not only affect the well-being of the mother, but may also have a significant impact on her ability to form a healthy bond with the newborn. In several studies, postpartum depressive symptomatology has been consistently associated with impaired MIB, from first days after delivery to 6 months postpartum [3, 4, 34–39]. Depressed mothers tend to be more hostile and irritable, show less warmth, be less engaged and play less with their infants [40]. Compared to depression, the impact of maternal anxiety on early postpartum MIB has received little research attention and studies have led to contradictory results. While some authors found that women’s postpartum anxiety was significantly related to lower postpartum MIB [38, 41, 42], there was also indication for an association with higher postpartum MIB [4]. The mixed findings of those studies might be due to underlying differences in the definition of maternal anxiety resulting in diverging assessment methods, assessment points and study populations. The current study regards depressive as well as anxiety symptoms as common mental health issues of postpartum women in relation to their MIB.

### The current study

In sum, more research is needed to obtain a detailed picture of the influences of maternal childhood maltreatment and postpartum mental health on early postpartum MIB. The present study aimed (a) to investigate the effect of maternal sexual, physical, and emotional abuse as well as physical and emotional neglect on postpartum MIB, and (b) to explore the influence of maternal postpartum depressive and anxiety symptoms on postpartum MIB. It was assumed that maternal childhood maltreatment would be associated with postpartum MIB impairment. No specific predictions were made about the differential effects of different types of maltreatment due to the explorative nature of the research question. Furthermore, it was hypothesised that higher levels of postpartum depressive and anxiety symptoms would be associated with more postpartum MIB impairment.

## Methods

### Participants and procedure

This study presents data from a cross-sectional study among women who gave birth to life-born babies between December 2013 and November 2014 at the Department of Obstetrics of the University of Leipzig (Germany). The study was conducted according to the Declaration of Helsinki and was approved by the local Ethics Committee. Eligible women were identified through an initial review of medical records and contacted within 16 weeks after delivery. If a phone number was available, women were called by a study member who explained the study. Women who agreed to take part in the study were sent study information, an informed consent sheet, the study questionnaire and a prepaid return envelope by postal mail or email. If no phone number was available, women were contacted by postal mail with complete study material given at this stage. In order to be included in the study, women had to be at least 18 years old at the time of assessment and had to state sufficient German reading and writing skills to answer the questionnaires Written informed consent was obtained from all participants before study inclusion.

### Measures

*Maternal childhood maltreatment* up to the age of 18 years was retrospectively assessed with the German version of the short form of the Childhood Trauma Questionnaire (CTQ) [43, 44]. The CTQ (28 items, 5-point Likert scale*, never true* to *very often true*, total score range 25–125 with higher scores reflecting higher maltreatment severity) covers five types of child maltreatment, namely sexual abuse (5 items), physical abuse (5 items), and emotional abuse (5 items) as well as physical neglect (5 items) and emotional neglect (5 items). In order to describe the study sample a dichotomous classification of each maltreatment type (present vs. absent) was used, based on the recommendations of Häuser et al. [45]. For main analyses the maltreatment forms were used as continuous variables. Reliability and validity of the German version of the CTQ were confirmed for community samples and comparable with the original version [46]. In the present sample, all subscales demonstrated good internal consistency (sexual abuse: α = .95; physical abuse: α = .78; emotional abuse: α = .87; emotional neglect: α = .90), except the subscale physical neglect (α = .40).

*Postpartum depression severity* during 14 days prior to the assessment was measured using the German version of the Revised Beck Depression Inventory (BDI-II) [47, 48]. The BDI-II (21 items, total score range 0–63 with higher scores indicating more severe depression symptomatology) has been widely used in both nonclinical and clinical samples. A cutoff of ≥ 14 was applied to identify participants with at least mild depressive symptoms. The German version of the BDI-II has shown satisfactory psychometric properties [49]. In the present sample, the internal consistency was high (α = .89).

*Postpartum anxiety* during the last 7 days prior to the assessment was measured using the anxiety subscale of the German version of the Symptom Checklist-90-Revised (SCL-90-R) [50, 51]. The anxiety subscale (10 items, 5-point Likert scale, *not at all* to *extremely*, subscale score range 0–40 with higher scores reflecting higher anxiety severity) is one of nine subscales, which assess current psychological distress. Participants scoring above the 75^th^ percentile were considered as cases with elevated anxiety symptoms. The German version has demonstrated good reliability [52]. In the present sample, the internal consistency was comparably high (α = .88).

*Postpartum MIB* was measured with the abridged German version of the Postpartum Bonding Questionnaire (PBQ-16) [3]. Brockington et al. [5] developed the questionnaire as a 25-item screening instrument for the diagnosis of MIB disorders, asking for maternal feelings about the infant (e.g., “I feel close to my baby”, “My baby irritates me”). The German validation study did not confirm the original four factor structure, but suggested one general factor called “bonding impairment” [3]. The PBQ-16 (16 items, 6-point Likert scale, *always* to *never*, total score range 0–80 with higher scores indicating more MIB impairment) has demonstrated good reliability. The internal consistency in the present sample was comparably high (α = .82).

*Sociodemographic control variables* including age, education, marital status and parity (number of births) were obtained through self-generated questions.

### Statistical analyses

Data analyses were performed using the Statistical Package for Social Sciences, version 24.0.0.2 (IBM® SPSS®). Pearson, respectively, point-biserial correlations between the outcome measure postpartum MIB and each of the other study variables were calculated. Afterwards, a hierarchical forced entry regression analysis was performed [53]. Three blocks of independent variables were entered hierarchically in order of their presumed theoretical importance and causal priority: (1) sociodemographic control variables, (2) maternal childhood maltreatment variables, and (3) maternal postpartum mental health variables. For the diagnosis of multicollinearity, the variance inflation factor was calculated, considering values above 5 as an indication of multicollinearity [54]. The assumption of independent errors was checked using the Durbin-Watson statistic. The regression model showed indication of heteroscedasticity. Hence, 95% bias-corrected and accelerated bootstrapped confidence intervals (95% BCaCI), based on 1000 bootstrap samples were calculated to obtain robust inferential statistics with regard to regression coefficients.

## Results

A total of 810 women participated in the study. For the current analyses, 36 women with multiple pregnancies were excluded. Due to missing values in the relevant study variables, another 49 cases (6.3%) were removed resulting in a final sample of 725 women. At the time of the assessment, the mean time interval to delivery was M = 8.09 weeks (SD = 3.13). Table 1 shows the sample characteristics. Participants’ age ranged from 18 to 43 years. The majority of women were of German nationality (95.6%), had ≥ 12 years of school education (hereinafter classified as “high education”) (72.4%), were married or in a partnership (79.6%) and had given birth to their first child (59.6%). Overall, 45.7% of women reported at least one type of childhood maltreatment with emotional neglect being reported most frequently (26.1%), followed by physical neglect (22.1%), and emotional abuse (17.8%). Sexual abuse was reported by 11.4% and physical abuse by 8.3%. Elevated postpartum depression symptoms were reported by 13% and elevated postpartum anxiety symptoms by 20.1% of women.

**Table 1:** Sample characteristics

Sociodemographic characteristics	
Age, M (SD)	30.58 (4.48)
Nationality: German, *n* (%)	691 (95.6)
Education: ≥ 12 years of school education, *n* (%)	525 (72.4)
Marital status: married or in a partnership, *n* (%)	577 (79.6)
Parity: primiparous, *n* (%)	432 (59.6)
Maternal childhood maltreatment	
Sexual abuse: CTQ subscale ≥ 6, *n* (%)	83 (11.4)
Physical abuse: CTQ subscale ≥ 8, *n* (%)	60 (8.3)
Emotional abuse: CTQ subscale ≥ 9, *n* (%)	129 (17.8)
Physical neglect: CTQ subscale ≥ 8, *n* (%)	160 (22.1)
Emotional neglect: CTQ subscale ≥ 10, *n* (%)	189 (26.1)
Maternal postpartum mental health	
Depression: BDI-II ≥ 14, *n* (%)	94 (13.0)
Anxiety: SCL-90-R anxiety subscale > 2, highest quartile, *n* (%)	146 (20.1)

Total sample *N* = 725 (percentages are calculated from valid cases); *M* mean; *SD* standard deviation

Table 2 presents the descriptive statistics for the study variables postpartum MIB, maternal childhood maltreatment and postpartum mental health. The mean sum scores for the questionnaires PBQ-16, CTQ, BDI-II and SCL-90-R were all low, indicating on average little postpartum MIB impairment, low maternal childhood maltreatment severity, low postpartum depression as well as low postpartum anxiety severity for the current sample of women.

**Table 2:** Descriptive statistics of study variables

	Min	Max	M	SE	SD
Dependent variable					
PBQ-16	0.00	38.00	6.64	0.20	5.35
Independent variables					
CTQ: sexual abuse	5.00	25.00	5.59	0.08	2.27
CTQ: physical abuse	5.00	24.00	5.61	0.07	1.88
CTQ: emotional abuse	5.00	25.00	7.04	0.12	3.34
CTQ: physical neglect	5.00	20.00	6.38	0.08	2.20
CTQ: emotional neglect	5.00	25.00	8.17	0.14	3.77
BDI-II	0.00	53.00	7.62	0.23	6.24
SCL-90-R: anxiety	0.00	30.00	1.86	0.14	3.67

*M* mean; *SE* standard error; *SD* standard deviation

Table 3 provides an overview of correlations between the outcome measure postpartum MIB and each of the other study variables. With the exception of age, parity, maternal physical abuse and physical neglect in childhood, all other variables showed significant positive correlations with postpartum MIB impairment.

**Table 3:** Bivariate correlations with postpartum MIB impairment

	PBQ-16
Sociodemographic control variables	
Age	.056
Education (1: ≥ 12 years of school education, 0: < 12 years of school education)	.142**
Marital status (1: married or in a partnership, 0: single, divorced or widowed)	.073*
Parity (1: primiparous, 0: multiparous)	.048
Maternal childhood maltreatment	
CTQ: sexual abuse	.115**
CTQ: physical abuse	.069
CTQ: emotional abuse	.162**
CTQ: physical neglect	.037
CTQ: emotional neglect	.212**
Maternal postpartum mental health	
BDI-II	.473**
SCL-90-R: anxiety	.289**

Pearson, respectively, point-biserial correlation coefficient with significance level, *p < .05; **p < .01

Results of the regression analysis are presented in Table 4. In step 1, the sociodemographic control variables explained significantly 2.8% of variance. In step 2, maternal childhood maltreatment showed a significant incremental explanation of variance of 7.5% beyond the sociodemographic control variables. The addition of maternal postpartum mental health in the final step led to an improvement of the model with significant changes in *R*^*2*^ of 18.7%. Overall, the final model explained 29% of variance in postpartum MIB (*F*_(11,713)_ = 26.49, *p* < .001) with more than half of the variance being explained by maternal postpartum mental health. The adjusted *R*^*2*^ indicated an explanation of variance of 27.9% which is, according to Cohen [55], a large effect size. There was no indication of multicollinearity and autocorrelation in the residuals. Regarding the standardized regression coefficients of the final model, six variables provided a significant contribution to postpartum MIB. Within the block of sociodemographic control variables, having a high education (*β* = .16, *p* < .001), being married or in a partnership (*β* = .08, *p* < .05), and giving birth to the first child (*β* = .09, *p* < .05) was associated with more postpartum MIB impairment. Within the block of maternal childhood maltreatment, physical neglect (*β* = −.09, *p* < .05) and emotional neglect (*β* = .25, *p* < .001) remained significant variables, even after adding postpartum depression and anxiety severity to the model, demonstrating an independent contribution of these maltreatment types to postpartum MIB. Higher severity of emotional neglect was associated with more postpartum MIB impairment, whereas higher severity of physical neglect was associated with lower postpartum MIB impairment. Within the block of postpartum mental health, only higher levels of postpartum depression (*β* = .49, *p* < .001), but not postpartum anxiety severity (*β* = −.03, *n.s.*) showed a significant association with more postpartum MIB impairment. In sum, the variables postpartum depression severity, maternal emotional neglect in childhood and education showed the strongest associations with postpartum MIB.

**Table 4:** Hierarchical forced entry regression analysis

	Postpartum MIB impairment (PBQ-16)		
	Step 1	Step 2	Step 3
	*β* [95% BCaCI]	*β* [95% BCaCI]	*β* [95% BCaCI]
Step 1: Sociodemographic control variables			
Age^a^	0.05 [−0.05, 0.17]	0.06 [−0.03, 0.18]	0.06 [−0.02, 0.16]
Education^b^ (1: ≥ 12 years of school education, 0: < 12 years of school education)	0.12* [0.52, 2.31]	0.15* [0.91, 2.72]	0.16* [1.15, 2.73]
Marital status^b^ (1: married or in a partnership, 0: single, divorced or widowed)	0.06 [−0.07, 1.78]	0.07* [0.04, 1.91]	0.08* [0.23, 1.78]
Parity^b^ (1: primiparous, 0: multiparous)	0.07 [−0.06, 1.59]	0.08* [0.10, 1.69]	0.09* [0.26, 1.62]
Step 2: Maternal childhood maltreatment			
Sexual abuse^a^ (CTQ)		0.08 [−0.11, 0.49]	0.01 [−0.19, 0.26]
Physical abuse^a^ (CTQ)		−0.05 [−0.44, 0.14]	−0.04 [−0.38, 0.13]
Emotional abuse^a^ (CTQ)		0.01 [−0.20, 0.24]	−0.10 [−0.37, 0.03]
Physical neglect^a^ (CTQ)		−0.12* [−0.49, −0.08]	−0.09* [−0.40, −0.04]
Emotional neglect^a^ (CTQ)		0.31* [0.24, 0.68]	0.25* [0.18, 0.53]
Step 3: Maternal postpartum mental health			
Depression^a^ (BDI-II)			0.49* [0.33, 0.52]
Anxiety^a^ (SCL-90-R)			−0.03 [−0.19, 0.12]
*R*^*2*^ (adjusted *R*^*2*^)	.028** (.022)	.103** (.091)	.290** (.279)
*∆R* ^*2*^		.075**	.187**

*β* standardized regression coefficient; 95% BCaCI 95% bias-corrected and accelerated bootstrapped confidence interval of *b* based on 1000 bootstrap samples; *∆R*^*2*^ change in *R*^*2*^; *p < .05 and 95% BCaCI did not include zero; **p < .001

^a^ continuous variable; ^b^ dichotomous variable

## Discussion

Considering the influence of early postpartum MIB on the child’s later social-emotional development, the current study aimed to examine maternal factors interfering with a healthy postpartum MIB. Study results suggest that maternal experiences of emotional neglect in childhood and postpartum depression were associated with more MIB impairment (i.e. poorer MIB), while maternal exposure to physical neglect was associated with less MIB impairment (i.e. better MIB) 2 months postpartum.

Overall, almost half of women reported a history of childhood maltreatment. The high prevalence in the current community sample is consistent with prior research [56]. Of the five analysed maltreatment types, both neglect forms were associated with postpartum MIB, even after adding maternal postpartum mental health. Some studies already discussed the potential of impaired MIB as a crucial mechanism by which maternal childhood maltreatment causes long-term disturbances in parenting [24–26]. However, the authors concluded that a trauma history alone may not determine poor MIB in the absence of co-occurring mental illness. In contrast, the current study suggests an independent effect of maternal childhood maltreatment on postpartum MIB. Whereas the majority of previous studies did not distinguish between different types of maltreatment, the current study explored diverse forms, taking into account their co-occurrence. Thus far, Farré-Sender et al. [27] have examined one single type of childhood maltreatment showing that maternal emotional abuse was a significant predictor of postpartum MIB impairment, which was not replicated in the current study. The analysis of only one type of childhood maltreatment could have led to an overestimation of its influence due to the common interrelations with other types of childhood maltreatment like emotional neglect [15]. Based on the current study it seems that maternal emotional neglect may account for the association between maternal emotional abuse and postpartum MIB impairment. Furthermore, the variations of the study findings might be due to different study populations. Farré-Sender et al. [27] conducted their study in a clinical population, whereas the current study was conducted in a general population.

Although the unique influences of childhood maltreatment types on postpartum MIB found in this study need to be replicated in future research, the results underline the impact of maternal neglect in childhood on later bonding with a mother’s own child. In particular, emotional maltreatment “creates a pervasive, enduring and influential context for child development with effects that persist into adulthood” ([57], p. 243). It seems plausible that growing up without a sensitive and responsive caregiver who provides emotional care hinders the development of an affectionate bond with the mothers’ own child. Indeed, in a study of the attachment of mothers to their unborn babies, maternal perceptions of more emotional warmth in their own childhood were associated with better prenatal attachment [58].

In contrast to emotional neglect, maternal physical neglect was associated with less postpartum MIB impairment. Given the low Cronbach’s alpha of the subscale physical neglect (CTQ) in the current study, this result should be interpreted with caution. One possible explanation could be the wish of the affected mothers to compensate their own negative experiences [58]. Furthermore, protective factors like a supportive romantic relationship could compensate the negative influence of a history of physical maltreatment on subsequent mother-infant relationship difficulties [59], and should therefore be examined additionally in future studies.

The current study not only differs from existing studies [24, 26] in the examination of diverse maltreatment types, but assessed postpartum mental health in terms of depression and anxiety, and not PTSD as common trauma-related mental disorder. However, a recent study showed a general negative effect of maternal childhood maltreatment experiences on postpartum mother-child-interactions in a non-clinical sample of women without PTSD or other psychiatric problems in their lifetime-history [60]. The comparison of different maltreatment groups showed that emotional abuse and neglect predicted more dyadic exchanges characterized by feelings of sadness in the mother than the experience of sexual or physical abuse.

Postpartum depression severity displayed the greatest association with postpartum MIB impairment, which adds to existing studies [4, 34, 35]. Specific symptoms of depression like an increased negative affect, emotional unavailability, and negatively biased cognitions may undermine the establishment of an intense emotional bond with the newborn [42, 61]. In addition, postpartum anxiety severity seemed to be positively related to postpartum MIB impairment, but this association disappeared when controlling for postpartum depression. This finding is in line with Tietz et al. [42], who demonstrated that it is not the diagnosis of anxiety disorder itself, but the presence of concurrent subclinical depressive symptoms which predicted lower MIB. Moreover, women with social phobia reported no differences in postpartum MIB compared to women without social phobia [62]. Future research should explore anxiety subgroup-specific effects on postpartum MIB, considering different diagnostic groups [63, 64].

The presented data underline the relevance of sociodemographic variables for postpartum MIB. Mothers, who were highly educated and married or in a relationship reported more postpartum MIB impairment. Although some studies have found no association between theses variables and MIB [36] or an inverse effect [39], the current findings were in line with previous research [3, 34, 35, 38, 65]. Reck et al. [3] argue, that the association between high maternal education and MIB impairment might not be due to an actual relationship, but driven by a reporting bias, i.e. highly educated women may report difficulties in their relationship with their infants more reflective and accurately. This explanation is supported by another study, which demonstrated that women with a higher tendency to respond in a socially desirable manner reported indeed less MIB impairment [38]. Kinsey et al. [35] concluded that the inverse socioeconomic social desirability bias could be due to a strong social disapproval of parents who feel negatively toward their infants, especially when they were less educated and single parent. In the current study, primiparous women demonstrated more postpartum MIB impairment, which was also observed by others. In the study of Kim et al. [66], first-time mothers feel less confident in parenting than experienced parents.

### Strengths and limitations

The major strength of the current study is the concurrent consideration of diverse types of maternal childhood maltreatment in relation to postpartum MIB in a large community-based sample, which gives a more comprehensive picture of relevant influences on bonding problems and provides specific cues for intervention. However, this study also has some limitations. The community sample showed a relatively high level of school education, limiting generalisability of results to other populations. The cross-sectional study design precludes any causal conclusions, with the exception of the influence of maternal childhood maltreatment on postpartum MIB. The exclusive use of self-report measures could have evoked a response bias due to social desirability. However, self-rating of postpartum MIB is significantly associated with observed parenting behaviour [24]. The assessment of maternal childhood maltreatment was based on the retrospective recall of adverse childhood experiences which is likely associated with measurement error [67]. Nonetheless, the German short form of the CTQ has proven to be a reliable and valid assessment instrument of childhood maltreatment [44, 46].

### Clinical implications

Although the reported findings are preliminary and need to be validated in prospective studies, they point to the need of providing short- and long-term psychological support for mothers with emotional neglect in childhood and postpartum depression as they may experience postpartum MIB impairment. Routine assessment of trauma history and psychopathology during prenatal visits is warranted to identify women at risk. Health care providers could then provide targeted counselling. There is already evidence for the effectiveness of early interventions. Participants of a psychoeducational program for pregnant women with a history of childhood maltreatment scored better in measures of postpartum MIB and depressive symptoms than participants who received usual care [68]. The implementation of trauma-informed care into maternity practice seems to have the potential to prevent adverse outcomes [69]. Furthermore, public initiatives could be created to promote access to mental health and parenting services as well as to destigmatise help-seeking behaviour for women who have been exposed to childhood maltreatment.

## Conclusions

The results of the current study suggest that maternal experiences of emotional neglect in childhood and postpartum depressive symptoms are associated with MIB impairment in the early postpartum period. Additional longitudinal research should address the course of trauma-related psychopathology and MIB as well as the underlying mechanisms during pregnancy and postpartum. Nonetheless, the current study demonstrates the importance of distinguishing among diverse types of maternal childhood maltreatment to better understand associations between traumatic experiences of women and postpartum MIB, which is necessary to provide optimum care for affected women.

## Data Availability

The datasets used for analysis during the current study are available from the corresponding author on request.

## Abbreviations

95% BCaCI: 95% bias-corrected and accelerated bootstrapped confidence interval
BDI-II: Revised Beck Depression Inventory
CTQ: Childhood Trauma Questionnaire
MIB: Mother-infant bonding
PBQ-16: Abridged German version of the Postpartum Bonding Questionnaire
PTSD: Posttraumatic stress disorder
SCL-90-R: Symptom Checklist-90-Revised
